# A 45 nm Stacked CMOS Image Sensor Process Technology for Submicron Pixel †

**DOI:** 10.3390/s17122816

**Published:** 2017-12-05

**Authors:** Seiji Takahashi, Yi-Min Huang, Jhy-Jyi Sze, Tung-Ting Wu, Fu-Sheng Guo, Wei-Cheng Hsu, Tung-Hsiung Tseng, King Liao, Chin-Chia Kuo, Tzu-Hsiang Chen, Wei-Chieh Chiang, Chun-Hao Chuang, Keng-Yu Chou, Chi-Hsien Chung, Kuo-Yu Chou, Chien-Hsien Tseng, Chuan-Joung Wang, Dun-Nien Yaung

**Affiliations:** Taiwan Semiconductor Manufacturing Company, No. 8, Li-Hsin Rd. 6, Hsinchu Science Park, Hsinchu 300, Taiwan; ymhuangd@tsmc.com (Y.-M.H.); jjsze@tsmc.com (J.-J.S.); ttwud@tsmc.com (T.-T.W.); fsguo@tsmc.com (F.-S.G.); wchsuo@tsmc.com (W.-C.H.); thtsengc@tsmc.com (T.-H.T.); ccliaot@tsmc.com (K.L.); cckuozf@tsmc.com (C.-C.K.); thchenqa@tsmc.com (T.-H.C.); wchianga@tsmc.com (W.-C.C.); chjuang@tsmc.com (C.-H.C.); kychoud@tsmc.com (K.-Y.C.); chchungu@tsmc.com (C.-H.C.); kychouc@tsmc.com (K.-Y.C.); chtsenga@tsmc.com (C.-H.T.); cjwang@tsmc.com (C.-J.W.); dnyaung@tsmc.com (D.-N.Y.)

**Keywords:** submicron pixel, image sensor, stacked CMOS image sensor, dark current, read noise, random telegraph noise, full well capacity, optical crosstalk

## Abstract

A submicron pixel’s light and dark performance were studied by experiment and simulation. An advanced node technology incorporated with a stacked CMOS image sensor (CIS) is promising in that it may enhance performance. In this work, we demonstrated a low dark current of 3.2 e^−^/s at 60 °C, an ultra-low read noise of 0.90 e^−^·rms, a high full well capacity (FWC) of 4100 e^−^, and blooming of 0.5% in 0.9 μm pixels with a pixel supply voltage of 2.8 V. In addition, the simulation study result of 0.8 μm pixels is discussed.

## 1. Introduction

Scaling down pixel size is absolutely necessary for high resolution imaging and quanta image sensors [1]. Recently, dual camera applications have become a major trend in the smartphone market [2], in which a small pixel size image sensor can be used as be a high resolution image sensor for the purpose of producing a zoomed image. In addition, phase detection auto focus function has developed. Among various pixel types of the function, dual photodiode phase detection auto focus also needs equivalently small pixel size [3].

**Figure 1:**
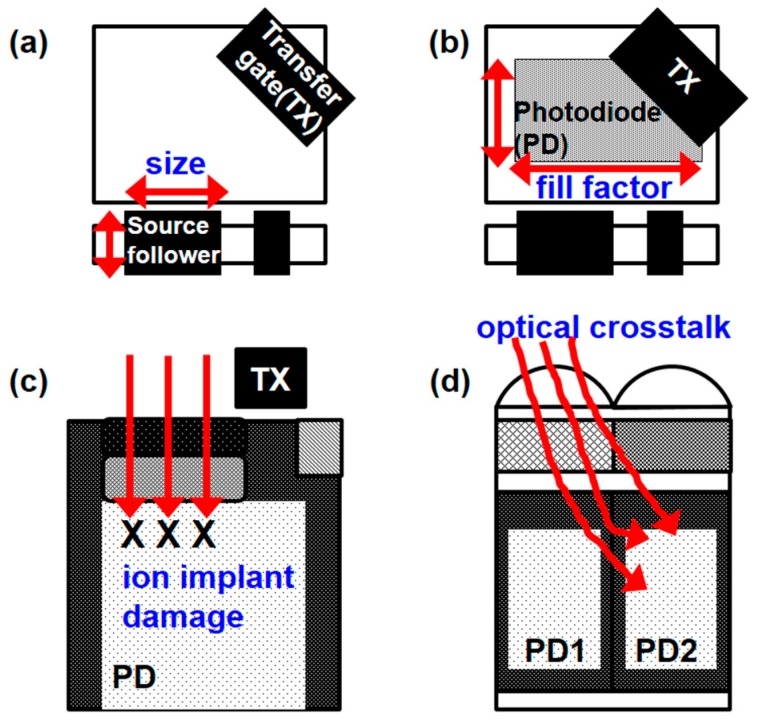
Challenges in submicron pixel generation. (**a**) Top view of pixel layout highlights the small size source follower; (**b**) top view of pixel layout indicating the small fill factor; (**c**) cross-sectional view of pixel depicting high ion implant damage induced by the high dose photodiode; (**d**) cross-sectional view of pixel showing high optical crosstalk.

However, sensor performances of the small pixel are generally inferior to those of previous generations with larger pixel sizes [4]. Major challenges in submicron pixel generation are shown in Figure 1. A small pixel does not have enough space for a large source follower device and a large photodiode. This induces higher source follower noise and a smaller fill factor. These in turn influence the dynamic range and the signal-to-noise ratio (SNR). Additionally, the required implant dosage is higher for smaller pixels, and a higher implant dosage will induce higher dark currents and white pixels due to ion implant damage. Moreover, crosstalk is significant due to a small pixel pitch. These two problems significantly affect image quality.

Backside illumination technology has been developed and has enabled drastic S/N improvement [5,6]. Stacked CMOS image sensor (CIS) chips enable a more flexible manufacturing process dedicated to image sensors [7]. Furthermore, an advanced node technology [8] with a stacked CIS might boost the light signal, reduce noise, and control the process variation caused by critical dimension fluctuations and mask overlay errors, which are more serious in submicron pixel generation.

In this paper, a silicon result of 0.9 μm pixels with well-balanced light and dark performance, making full use of a highly manufacturable 45 nm advanced technology with a stacked CMOS image sensor [9], and 0.8 μm pixel simulation data are presented.

## 2. A 45 nm Stacked CMOS Image Sensor

**Figure 2:**
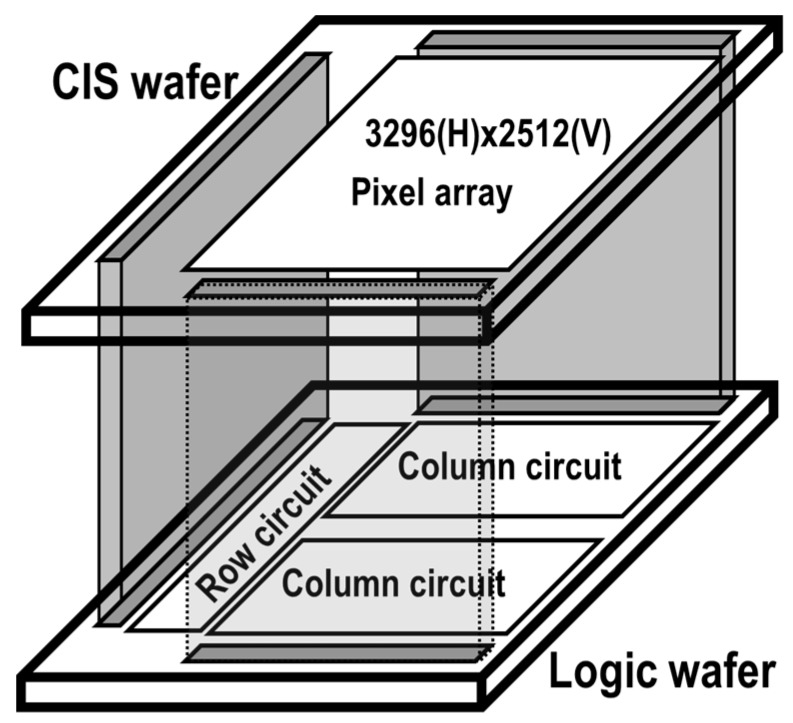
Block diagram of 45 nm stacked CIS test vehicle.

The test chip architecture is an 8-mega-pixel (3296(H) × 2512(V)) raw data output CIS test vehicle. The block diagram of the vehicle is illustrated in Figure 2. The image sensor consists of two silicon layers. The top wafer comprises a pixel array and the bottom wafer comprises a read out circuit. Since a stacked CIS chip has a small camera module and a flexible design, a column level bonding stacked CIS architecture was adopted. 

There are two possibilities in terms of pixel device placement in a stacked CIS chip. One is to place it on a CIS wafer, and the other is to place it on a logic wafer. Table 1 summarizes the pros and cons of these two choices. Pixel devices on a CIS wafer lead to a highly flexible pixel device process but a lower full well capacity (FWC) due to a lower fill factor. On the other hand, pixel devices on a logic wafer lead to a higher FWC and have a simple pixel structure, consisting only of a transfer gate and a photodiode, but they have a lower conversion gain, which leads to higher noise, since the wiring between the logic wafer and the CIS wafer has some parasitic capacitance, and it is not negligible.

**Figure 3:**
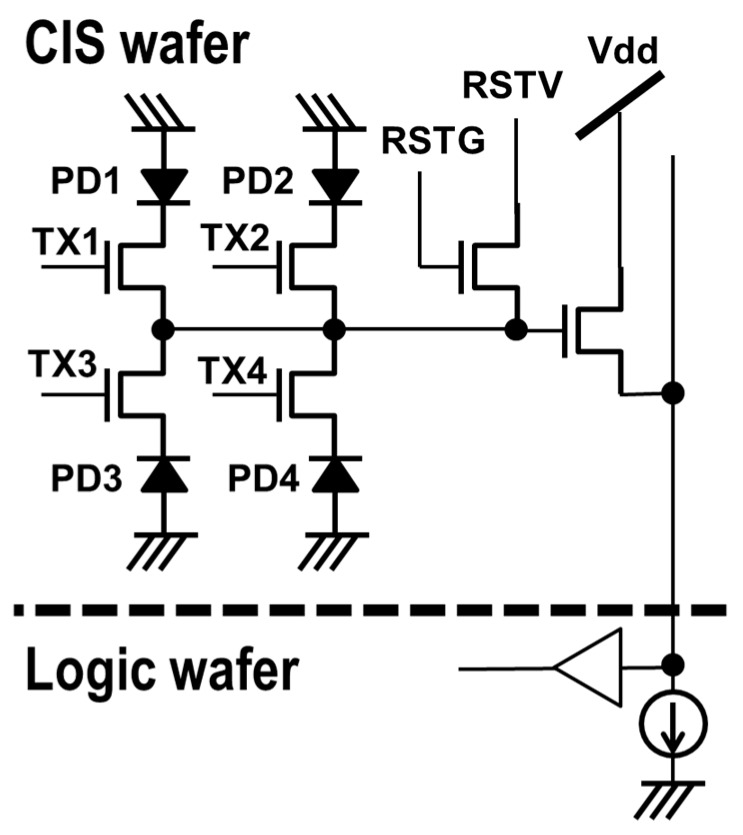
A unit pixel circuit and device partition.

Taking into account the overall pixel performance, all pixel devices were placed on a CIS wafer, as shown in Figure 3. Since pixel devices were kept on a CIS wafer, advanced 45 nm technology was desired in terms of its low noise and high fill factor. The pixel architecture adopted a 2 × 2 shared 4-transistor without row-select, and the pixel unit cell size was 0.90 μm.

The processed CIS wafer was bonded with a logic wafer, followed by backside illumination process including thin down, anti-reflection coating, a color filter, and a micro-lens array process [10].

## 3. Experimental Result

### 3.1. Low Noise Source Follower Device

An input referred noise in a conventional CIS is represented as follows [11]:

(1) $$N\;input\;referred=\sqrt{N\;pixel^{2}+{\left(\frac{V\;circuit+V\;source\;follower}{Av\cdot CG}\right)}^{2}}$$

(2) $$V\;source\;follower\propto \frac{N\;trap\;density}{Cox\cdot W\cdot L}$$

where *N pixel*, *V circuit*, and *V source follower* represent the noise generated at the pixel and the noise voltage generated in the circuit and source follower, respectively, *Av* is the circuit gain, *CG* is the conversion gain, *Cox* is the source follower gate capacitance, *W* is the source follower device width, *L* is the source follower device length, and *N trap density* is the trap state density.

It is clear from Equation (1) that increasing conversion gain is essential for reducing noise. A 45 nm design is beneficial for high conversion gain because the design rules are tighter than those of previous 65 nm node technology. The silicon result showed a conversion gain of 0.90 μm pixels reaching as high as 120 μV/e^−^.

After making an effort to reduce circuit noise, the signal chain noise was almost equivalent to the source follower flicker noise in the pixel array block, which was proportional to trap density and inversely proportional to gate capacitance, transistor width, and length.

Lithography capability is a key process element in this submicron pixel development. A 193 nm ArF immersion lithography was used for critical layers; as a result, the fill factor of 0.90 μm pixel increased by 20% with respect to the 65 nm technology. With the tightened design rules, the source follower device gate area can be maximized in a given small area in order to decrease random noise and random telegraph noise (RTN) [12]. The scaling of gate oxide thickness is also effective for random noise reduction [13].

**Figure 4:**
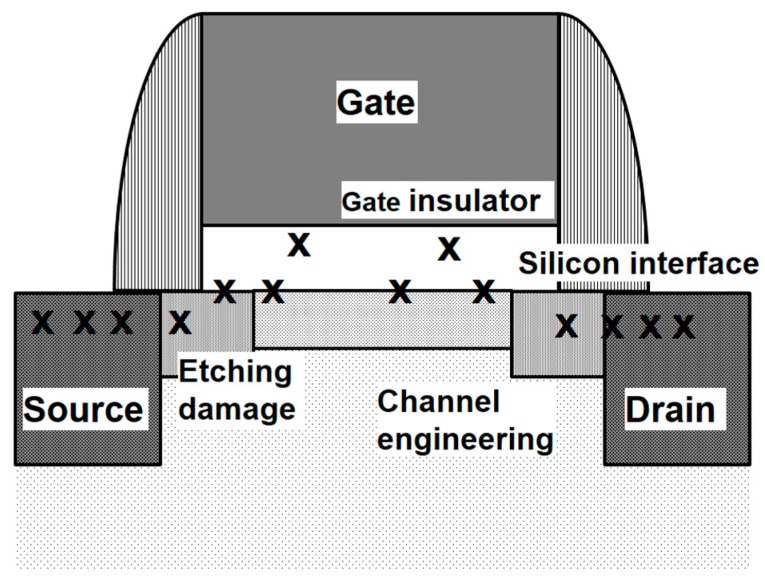
Defects that influence the source follower device noise.

Traps influencing the source follower noise exist in the gate insulator, at the silicon interface, and in bulk, as shown in Figure 4. Thanks to the dedicated CIS wafer process, related processes, for instance, minimizing etching damage, eliminating dangling bonds, and device channel engineering, have been fully optimized [14].

**Figure 5:**
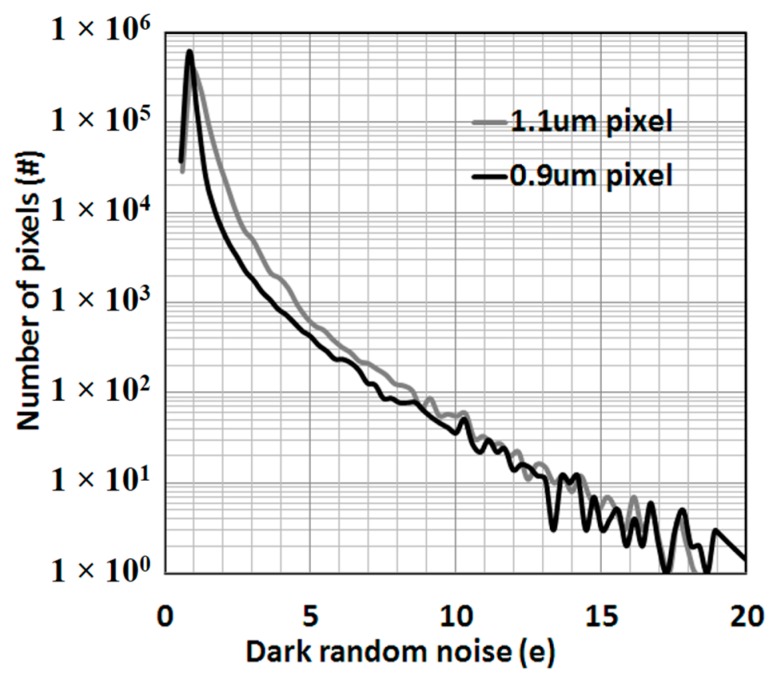
Statistical read noise distributions of the 0.9 μm pixel and the 1.1 μm pixel at an analog gain of 18 dB.

As a result, one of the key performance indexes, read noise, was reduced to 0.90 e^−^·rms at an analog gain of 18 dB, as shown in Figure 5, where the gray color indicates a 1.1 μm pixel with W = 0.2 μm and L = 0.8 μm, and the black color indicates a 0.9 μm pixel with W = 0.2 μm and L = 0.6 μm. Random telegraph noise, which contributes to the tail part of the distribution, also improved in spite of the smaller source follower device.

### 3.2. Low Dark Current Pixel

**Figure 6:**
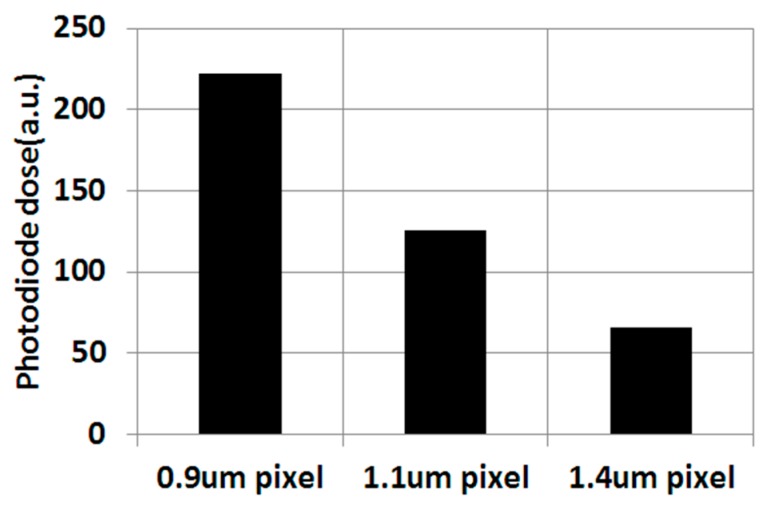
Pixel generation vs. the photodiode implant dose.

Another process integration challenge in the submicron pixel is the dark current reduction. Unlike logic transistors, an image sensor basically follows a constant voltage scaling law [15]. In the constant voltage scaling law, let *K* be a scaling factor which is greater than 1, doping concentration is proportional to *K* squared, and electric field is proportional to *K*. Figure 6 shows the necessary photodiode doping concentration. As expected, a smaller pixel requires a higher photodiode dosage, and a higher ion implant dosage induces more higher ion implant damage in bulk silicon, in addition to a higher electric field.

Dark current and white pixels are functions of defect density and electric field, so reducing these is fundamental.

**Figure 7:**
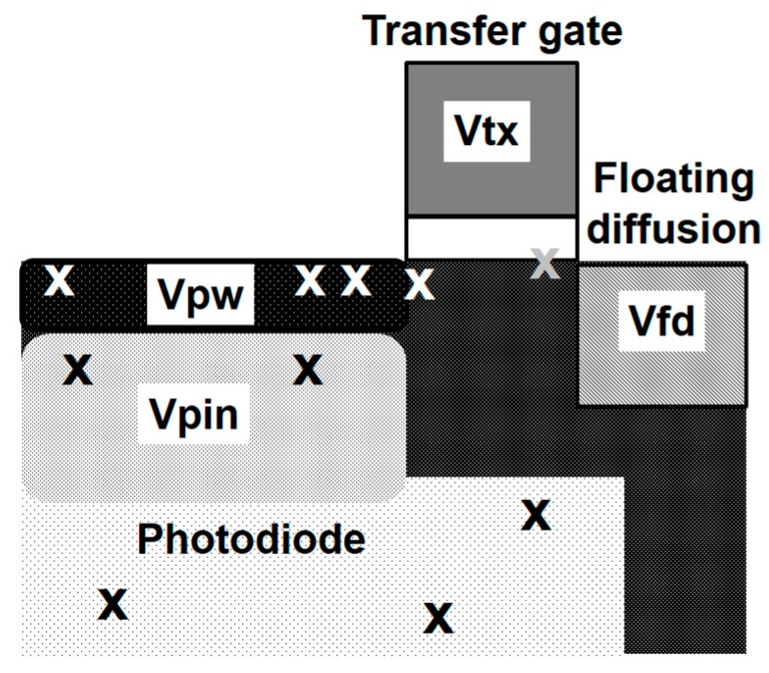
Dark current sources marked by “X” and pixel biases.

It is well known that defect located at the transfer gate edge is a main source of dark current and white pixels (Figure 7) [16]. Concerning defect density reduction, key process conditions such as ion implantation and annealing steps were carefully optimized to minimize every kind of defect and to recover damages.

### 3.3. Pixel Design and Low Dark Current Pixel

The electric field is decreased by device engineering. Reducing floating diffusion node bias (Vfd) works to reduce dark current due to electric field relaxation. However, this degrades anti-blooming due to a lower overflow potential [17,18]. Therefore, a new pixel structure was developed.

**Figure 8:**
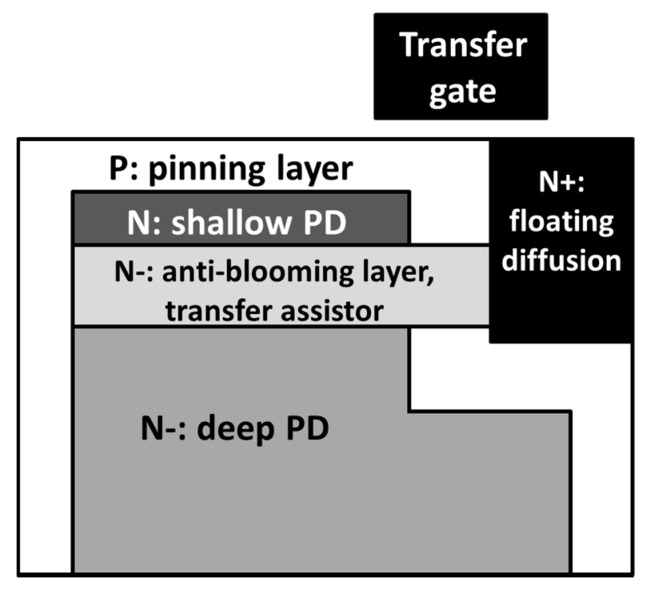
Schematic drawing of pixel design concept.

An additional *N*-type layer was interposed between a shallow photodiode (PD) and a deep photodiode, which also extended to the floating diffusion region (Figure 8). The floating diffusion node bias can be decreased by increasing the additional *N*-type layer dosage, and anti-blooming performance is not affected.

**Figure 9:**
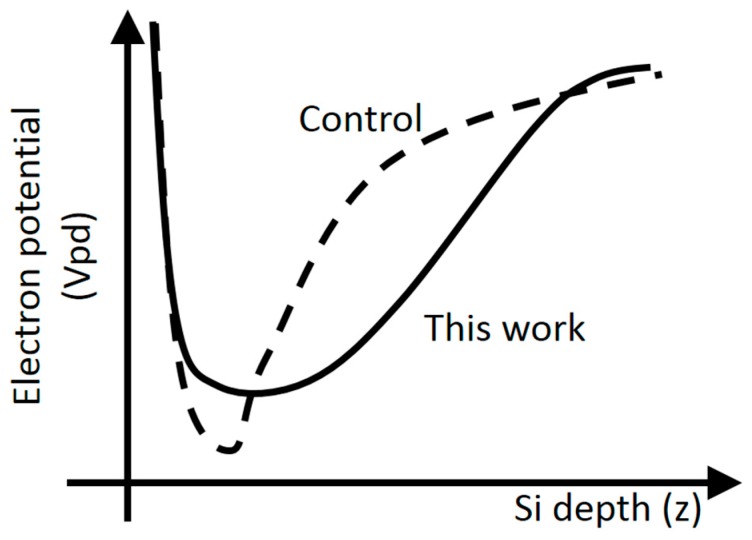
Photodiode potential profile. Dashed line is based on control pixel, and solid line is based on the pixel in this experiment.

This additional n-type layer is adequate to improve image lag as well. Thanks to the larger image lag margin, by utilizing a deep silicon area as charge storage, the pinning voltage (Vpin) of the photodiode can be decreased while the same FWC is maintained, as shown in Figure 9. Lower pinning voltage also works to reduce dark current.

**Figure 10:**
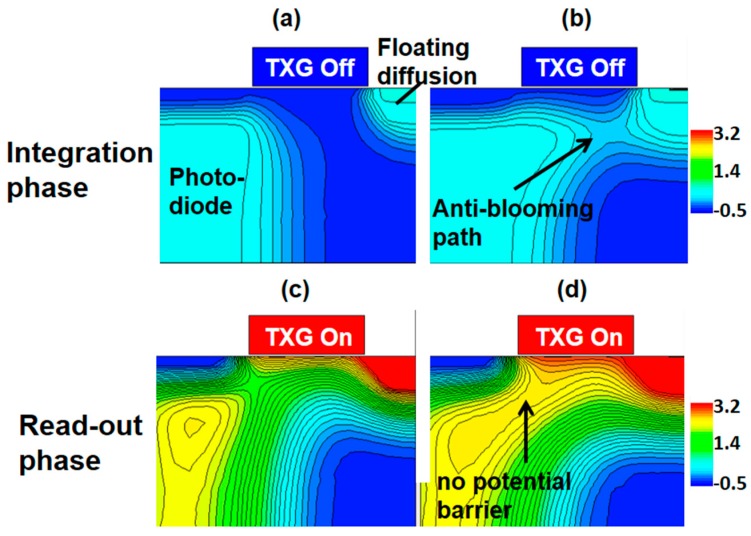
3D TCAD simulations of the transfer device structure showing electrostatic potential contours. (**a**) The control pixel during the integration phase; (**b**) the pixel in this experiment during the integration phase; (**c**) the control pixel during the read out phase; (**d**) the pixel in this experiment during the read out phase.

As shown in Figure 10, it is evident from the three-dimensional technology computer aided design (TCAD) device simulation results that an anti-blooming path was clearly made, and charge transfer capability improved as well.

**Figure 11:**
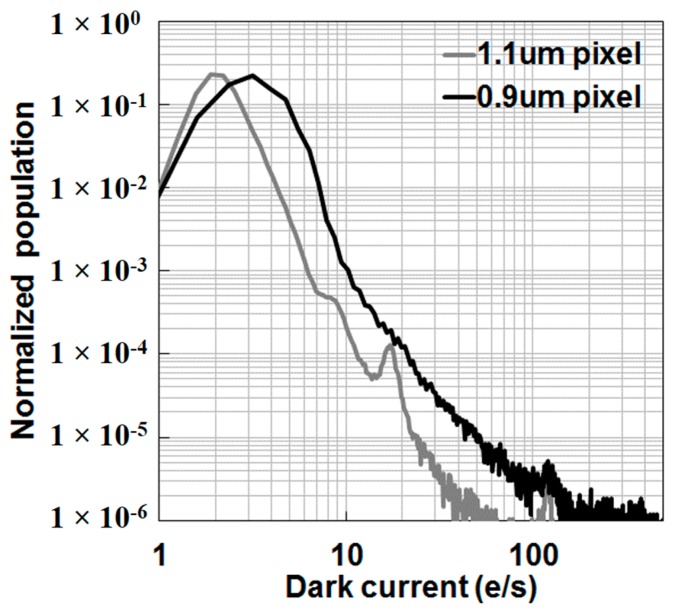
Dark current histograms at 60 °C of the 0.9 μm pixel and the 1.1 μm pixel. The gray color indicates the 1.1 μm pixel, and the black color indicates the 0.9 μm pixel.

A histogram of the individual pixel dark current at 60 °C is shown in Figure 11. The dark current peak at 60 °C corresponds to 3.2 e^−^/s for the 0.9 μm pixel, and the dark current distribution of the 0.9 μm pixel is close to that of the 1.1 μm pixel in spite of the higher photodiode dose.

### 3.4. Anti-Blooming Pixel

**Figure 12:**
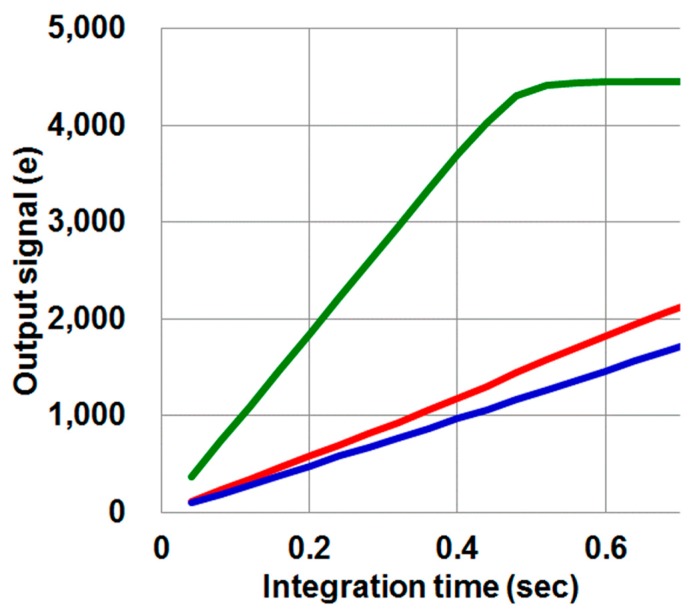
Light response curve of the 0.9 μm pixel.

Figure 12 shows a light response curve of 0.9 μm pixels. The green color is the green channel, and red is the red channel, and blue is the blue channel. As the TCAD device simulation results show, even after the green channel signal saturated, the adjacent channels, the red and blue signals, were not distorted. From this fact we can conclude that blooming was 0.5% [19]. This data also shows a measured linear FWC of 4100 e^−^.

### 3.5. Low Crosstalk Pixel

**Figure 13:**
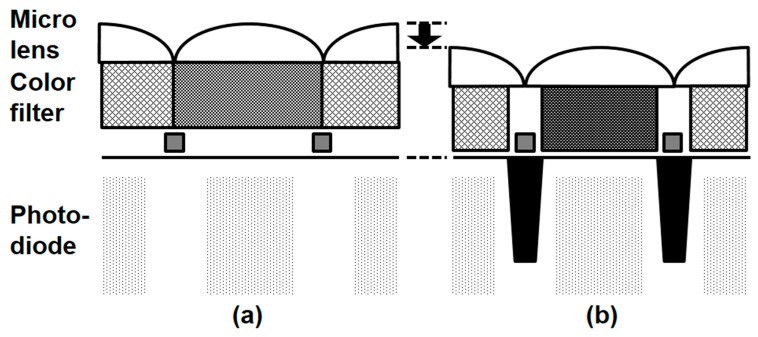
Schematic cross-sectional views of pixel. (**a**) Control pixel; (**b**) crosstalk-improved pixel.

Optical crosstalk improvement is mandatory in submicron small pixels [20], so we developed optical stack thinning and cross talk suppression techniques. Figure 13 shows a cross-sectional view of the pixel in the backside portion. The optical stack height, defined by the distance between the backside silicon surface and the top of the micro lens, decreased by 10%. Deep trench isolation (DTI) technology was developed to suppress optical crosstalk without sacrificing dark performance in parallel. In addition, a new color filter material was used to improve the SNR10 index.

**Figure 14:**
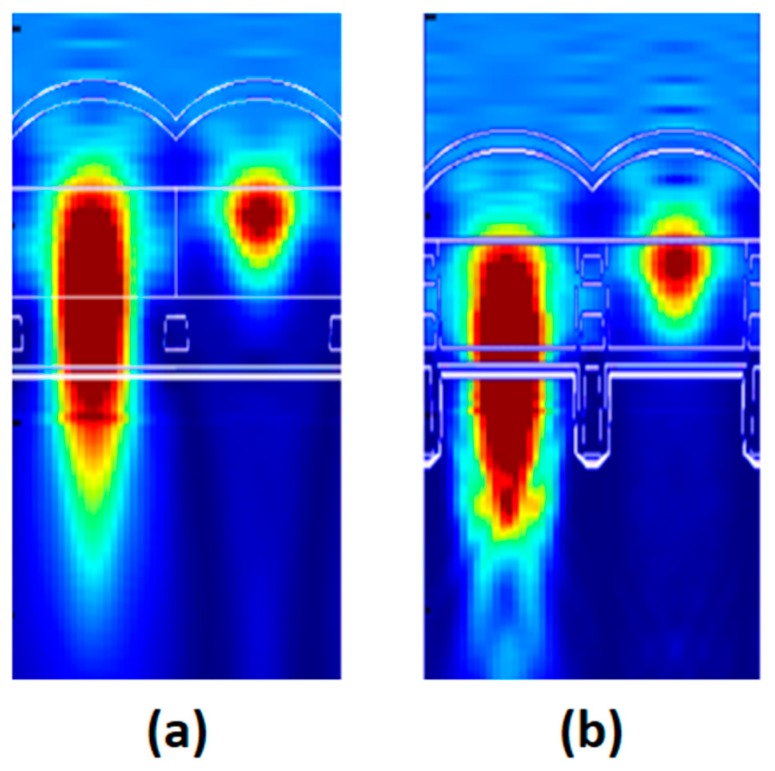
Optical simulation results at an incident wavelength of 530 nm. (**a**) Control pixel; (**b**) crosstalk-improved pixel.

In order to design an optical structure, we performed a three-dimensional finite difference time domain optical simulation. Based on the optical simulation, a deep trench isolation structure and material, as well as the curvature of the micro-lens, were determined. Figure 14 shows the optical simulation results. The simulation result of the improved pixel exhibits greater light-gathering capability.

**Figure 15:**
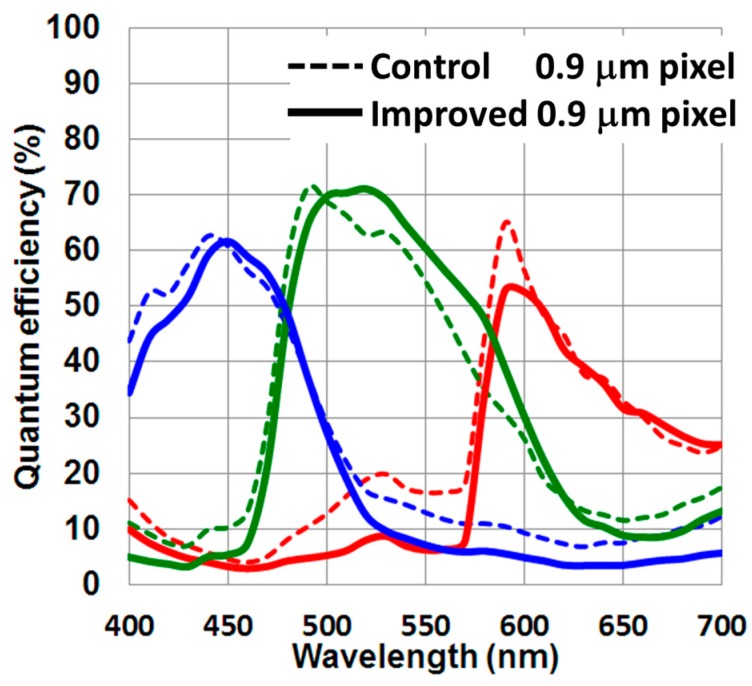
Measured quantum efficiency spectra of 0.9 μm pixels. Dashed line is the control 0.9 μm pixel, and the solid line is the improved 0.9 μm pixel.

Taking into account the optical simulation study results, we made a silicon sample. Obtained quantum efficiency (QE) spectra of the 0.9 μm pixels are shown in Figure 15. Optical crosstalk was greatly suppressed, and the blue and red responses slightly decreased due to a slightly smaller aperture area of the grid structure. Improving the green channel’s quantum efficiency is realized by a newly developed color filter material.

**Figure 16:**
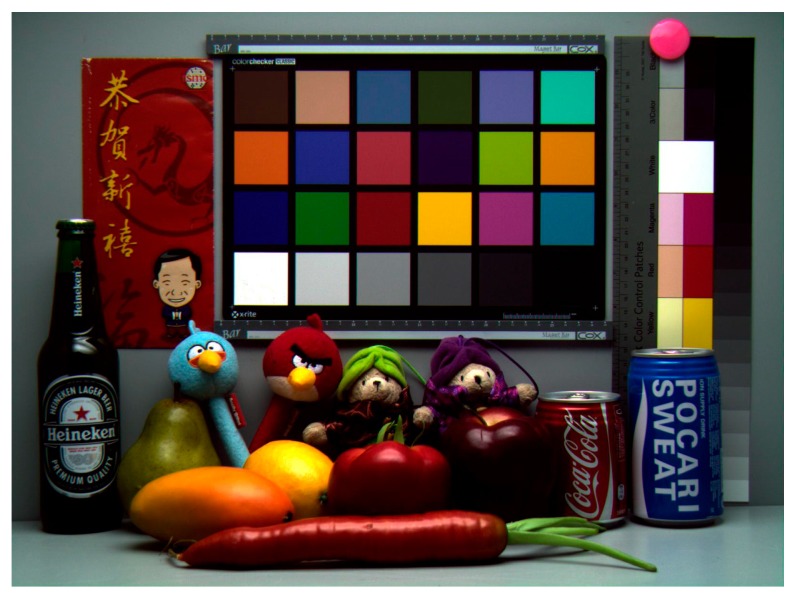
A sample color image taken with the 0.9 μm pixel manufactured in the 45 nm stacked CIS.

A sample color image taken with the 0.9 μm pixel in the 45 nm stacked CIS process technology is shown in Figure 16. There was no dead line, nor any defects, so this process is robust. Table 2 gives a summary of pixel performance. The process technology consists in 45 nm 1-poly 4-metal (1P4M) stacked CIS. The pixel supply voltage was 2.8 V. Image lag was less than 1 e. Photo response non-uniformity was 0.90%, and the QE at the green peak was 71%.

## 4. 0.8 μm Pixel Generation

The 0.9 μm pixel performance met market requirements, so we then decided to study 0.8 μm pixel generation. Assuming a 0.8 μm pixel uses a similar pixel structure and architecture, we estimated the performance by simulation and calculation.

First, we considered pixel noise and pixel RTN. Regarding RTN, source follower channel length is a key factor, and the length should be greater than 0.5 μm to efficiently suppress RTN [14]. Our pixel layout study confirmed that a more advanced node technology can provide a source follower device with a channel length greater than 0.5 μm in 0.8 μm pixels.

The optical cross-talk problem can be improved by deepening the DTI. In addition, a further thinning down of the optical stack height is still effective.

The most serious problem is still a low FWC. If the pixel size shrinks down from 0.9 to 0.8 μm based on the same process technology, FWC will be about 2800 e^−^, and photon noise will limit the image quality [21]. To maintain a reasonable image quality, the FWC needs at least 3000 e^−^. A simplified form of FWC can be expressed as

(3) FWC∝∫Cpd(z)·(Vpd(z)−Vovf)dz

where *z* is the silicon depth, *Cpd(z)* and *Vpd(z)* are the photodiode capacitance at a silicon depth of *z* and the depleted photodiode potential at a silicon depth of *z*, respectively, and *Vovf* is the overflow potential.

**Figure 17:**
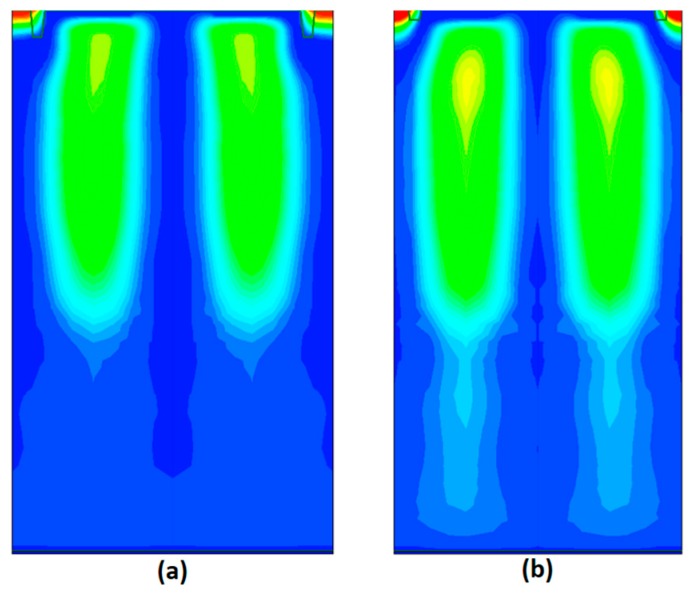
Three-dimensional TCAD simulations of photodiode showing electrostatic potential contours. (**a**) Two 0.9 μm pixels; (**b**) two 0.8 μm pixels.

*Vovf* is determined to fulfill anti-blooming criterion and cannot be changed. *Cpd(z)* can be increased by tightening the pixel-to-pixel isolation design rule. The simulated FWC with a tighter design rule then increases from 2800 to 3500 e^−^, rendering the quality of an image possibly acceptable. Comparisons between 0.9 μm pixel and 0.8 μm pixel can be made by studying Figure 17, in which the 0.8 μm pixel shows narrower pixel-to-pixel isolation.

Further improvement can be made by designing a vertically extended photodiode potential structure, or by simply increasing the pixel supply voltage, which would allow for the use of a higher *Vpd(z)*. It should be noted that the suppression of dark current and white pixel caused by a higher electric field in a photodiode must be taken into account.

## 5. Conclusions

The 45 nm advanced technology is desirable for submicron pixel generation due to tighter design rules and higher controllability for process variation. In addition, the flexibility of the stacked CIS process improves pixel performance. A novel 0.9 μm pixel with well-balanced light and dark performances, making full use of a highly manufacturable 45 nm advanced technology with a stacked CIS, is presented here. A low dark current of 3.2 e^−^/s at 60 °C, an ultra-low read noise of 0.90 e^−^·rms, a high FWC of 4100 e^−^, and blooming of 0.5% are demonstrated in a 0.9 μm pixel with a pixel supply voltage of 2.8 V. A simulation study of 0.8 μm pixel indicates that more advanced node technology with tightened pixel design rules lead to acceptable pixel performances. This technology offers image sensors a high resolution, superior low light imaging, and small chip size features.

## Figures and Tables

**Table 1:** Pros and cons of two different methods of pixel device placement.

Choice	PROS	CONS
Pixel devices on CIS wafer	High conversion gain	Low fill factor
	Dedicated pixel device process	
Pixel devices on logic wafer	High fill factor	Low conversion gain
	Dedicated photodiode process	

**Table 2:** Sensor characteristics of the 0.9 μm pixel.

Process Technology	45 nm 1P4M Stacked CIS
Pixel size	0.90 μm
Pixel supply voltage	2.8 V
Conversion gain	120 μV/e^−^
Dark current at 60 °C	3.2 e^−^/s
White pixel counts with dark current of >200 e^−^/s at 60 °C	679 ppm
Read noise at 18 dB	0.90 e^−^·rms
Full well capacity	4100 e^−^
Blooming	0.5%
Image lag	<1 e^−^
Photo response non-uniformity	0.9%
Quantum efficiency at green peak	71%
